# Impact of Oxidative Stress and Protein S-Glutathionylation in Aortic Valve Sclerosis Patients with Overt Atherosclerosis

**DOI:** 10.3390/jcm8040552

**Published:** 2019-04-24

**Authors:** Vincenza Valerio, Veronika A. Myasoedova, Donato Moschetta, Benedetta Porro, Gianluca L. Perrucci, Viviana Cavalca, Laura Cavallotti, Paola Songia, Paolo Poggio

**Affiliations:** 1 Centro Cardiologico Monzino IRCCS, Unit for the Study of Aortic, Valvular and Coronary Pathologies, 20138 Milan, Italy; vincenza.valerio@ccfm.it (V.V.); veronika.myasoedova@ccfm.it (V.A.M.); donato.moschetta@ccfm.it (D.M.); paola.songia@ccfm.it (P.S.); 2 Università degli Studi di Napoli Federico II, Dipartimento di Medicina Clinica e Chirurgia, 80131 Napoli, Italy; 3 Centro Cardiologico Monzino IRCCS, Unit of Metabolomics and Cellular Biochemistry of Atherothrombosis, 20138 Milan, Italy; benedetta.porro@ccfm.it (B.P.); viviana.cavalca@ccfm.it (V.C.); 4 Centro Cardiologico Monzino IRCCS, Unit of Vascular Biology and Regenerative Medicine, 20138 Milan, Italy; gianluca.perrucci@ccfm.it; 5 Centro Cardiologico Monzino IRCCS, Cardiac Surgery Unit, 20138 Milan, Italy; laura.cavallotti@ccfm.it

**Keywords:** calcific aortic valve disease, coronary artery disease, glutathione homeostasis, endothelial cells, endothelial to mesenchymal transition

## Abstract

Aortic valve sclerosis (AVSc) is characterized by non-uniform thickening of the leaflets without hemodynamic changes. Endothelial dysfunction, also caused by dysregulation of glutathione homeostasis expressed as ratio between its reduced (GSH) and its oxidised form (GSSG), could represent one of the pathogenic triggers of AVSc. We prospectively enrolled 58 patients with overt atherosclerosis and requiring coronary artery bypass grafting (CABG). The incidence of AVSc in the studied population was 50%. The two groups (No-AVSc and AVSc) had similar clinical characteristics. Pre-operatively, AVSc group showed significantly lower GSH/GSSG ratio than No-AVSc group (*p* = 0.02). Asymmetric dimethylarginine (ADMA) concentration was significantly higher in AVSc patients compared to No-AVSc patients (*p* < 0.0001). Explanted sclerotic aortic valves presented a significantly increased protein glutathionylation (Pr-SSG) than No-AVSc ones (*p* = 0.01). In vitro, inhibition of glutathione reductase caused β-actin glutathionylation, activation of histone 2AX, upregulation of α2 smooth muscle actin (*ACTA2*), downregulation of platelet and endothelial cell adhesion molecule 1 (*PECAM1*) and cadherin 5 (*CDH5*). In this study, we showed for the first time that the dysregulation of glutathione homeostasis is associated with AVSc. We found that Pr-SSG is increased in AVSc leaflets and it could lead to EndMT via DNA damage. Further studies are warranted to elucidate the causal role of Pr-SSG in aortic valve degeneration.

## 1. Introduction

Calcific aortic valve stenosis (CAVS) is a slow, progressive, multi-factorial disease characterized by dystrophic calcification of the aortic valve [1]. The initial phase, known as aortic valve sclerosis (AVSc), includes non-uniform thickening of the aortic leaflets, whereas advanced stages, named aortic valve stenosis (AS), are associated with impaired leaflet motion, causing restricted aortic valve area and thus resistance to blood flow [2,3]. To date, it is estimated that, in the general population, almost 30% of people over 65 years of age present AVSc with a drastically increased prevalence to develop severe AS, myocardial infarction or stroke [4,5]. Indeed, growing evidence suggests that AVSc is deeply associated with cardiovascular events [6,7]. However, little is known regarding its initial pathogenic mechanisms. Recent meta-analyses evaluating subclinical atherosclerosis revealed that AVSc is associated with increased carotid intima-media thickness, carotid plaques, coronary plaques, altered flow-mediated dilation, pulse wave velocity and augmentation index [6,7]. Endothelial dysfunction could represent the initial trigger of aortic leaflet structural deterioration. Indeed, endothelial dysfunction is fundamentally associated with several vascular conditions such as hypertension, diabetes and atherosclerosis [8].

One of the causes of endothelial dysfunction is the imbalance of nitric oxide (NO) biosynthesis and reactive oxygen species (ROS) production, called oxidative stress [9]. The main endogenous redox homeostasis mechanism is represented by the nucleophile glutathione (GSH) system since it can counteract ROS deleterious effects [8]. The ratio between the reduced (GSH) and the oxidized (GSSG) forms of glutathione is a recognized index of oxidative stress [10]. Interestingly, the dysregulation of glutathione homeostasis has been associated with several cardiovascular disorders such as hypertension and atherosclerosis [11]. In addition, several studies have shown a direct involvement of this dysregulation in cardiovascular disease progression [12,13,14].

When glutathione homeostasis is altered, endothelial and/or smooth muscle cells are subjected to the deleterious effects of ROS, which react with exposed cysteine residues of proteins. These susceptible cysteines can interact, in turn, with GSH and/or GSSG giving rise to reversible protein S-glutathionylation (Pr-SSG) [15,16].

Pr-SSG could occur in several structural and functional proteins, modifying their functions [17]. In particular, Pr-SSG is emerging as a critical signalling mechanism in cardiovascular diseases, possibly contributing to their aetiology [18]. Thus, we have hypothesized that altered GSH homeostasis could be a trigger for AVSc development via endothelial-to-mesenchymal transition (EndMT).

## 2. Patients and Methods 

### 2.1. Patient Population

Fifty-eight patients that underwent coronary artery bypass grafting (CABG) were enrolled in the study between January and June 2011. Pre-operative inclusion criteria were isolated surgical myocardial revascularization, elective surgery, age more than 18 years old, ejection fraction >30% and normal sinus rhythm. Exclusion criteria were prior cardiac surgery, rheumatic heart disease, endocarditis, active malignancy, chronic liver and kidney diseases, calcium regulation disorders (hyperparathyroidism, hyperthyroidism and hypothyroidism) and chronic or acute inflammatory states (sepsis, autoimmune disease and inflammatory bowel disease). 

C-reactive protein levels were collected from the clinical routine evaluation: pre-operatively, at 3rd day, at 5th day and at discharge (postoperative day 6th−7th). Blood samples for research purposes were obtained the day before surgery.

The Institutional Review Board and Ethical Committee of Centro Cardiologico Monzino (IRCCS) approved the study. Written informed consent to participate in this prospective observational study was obtained from all enrolled patients. The study protocol conforms to the ethical guidelines of the 1975 Declaration of Helsinki.

### 2.2. Echocardiographic Evaluation

Pre-operative echocardiographic evaluation with M-mode, two-dimensional and pulsed, continuous and colour-flow Doppler capabilities were performed for all CABG patients. Morphology and function of the aortic valve were assessed and the presence of AVSc was identified as non-uniform thickening with or without spotty calcified areas of the aortic valve leaflets without a significant transvalvular gradient (maximum aortic velocity <2.5 m/s) [19]. AVSc identification was performed, in a blind fashion way, by an expert clinician (VAM) and an expert cardiac surgeon (LC) resolved the borderline cases.

### 2.3. Blood Sampling and Biochemical Measurements

Whole blood: 6 mL of peripheral blood sample was drawn from patients and controls while fasting, into tubes containing EDTA (9.3 mM; Vacutainer Systems, Becton Dickinson, Franklin Lakes NJ, USA) kept on ice. 250 uL of whole blood was immediately precipitated with 250 uL of 10% trichloroacetic acid (Sigma-Aldrich, Darmstadt, Germany) plus 1 mM EDTA solution. Samples were stored at −80 °C until analysis.

Plasma EDTA: anti-coagulated blood was centrifuged at 1700 g for 10 min at 4 °C within 30 min after being drawn. Plasma was separated and aliquots were stored at −80 °C until analysis.

### 2.4. Glutathione Measurements

Reduced (GSH) and oxidized glutathione (GSSG) forms were determined in whole blood by liquid chromatography-tandem mass spectrometry (LC-MS/MS) method. The separation of analytes was conducted on a Luna PFP analytical column (100 × 2.0 mm, 3 µm, Phenomenex) eluted at 35 °C under isocratic conditions at 250 µL/min by 1% methanol in 0.75 mM ammonium formate adjusted to pH 3.5 with formic acid. LC-MS/MS analysis was performed using an Accela HPLC (high performance liquid cromatography) system coupled with a triple quadrupole mass spectrometer TSQ Quantum Access (Thermo Fisher Scientific, Waltham, MA, USA) using electrospray ionization source and multiple reaction monitoring (MRM) in positive mode. Data were obtained after comparison with calibration curves using GSH and GSSG pure standard solutions (Sigma-Aldrich, Darmstadt, Germany). The intra- and inter-CVs (%) obtained with standard samples were <5% for both the analytes. The limits of detection were 0.031 µmol/L for GSH and 0.008 µmol/L for GSSG. Levels of GSH and GSSG were corrected for haemoglobin (Hb) and expressed as µmol/g Hb.

### 2.5. Asymmetric Dimethylarginine Measurements

The assessment of asymmetric dimethylarginines (ADMA) was performed by LC-MS/MS using a target metabolomic approach. Briefly, the chromatographic analysis was conducted on a Luna HILIC (hydrophilic interaction liquid chromatography) analytical column (50 × 2.0 mm, 3 µm, Phenomenex, Torrance, CA, USA). The mobile phases consisted of aqueous 1.5 mM ammonium formate (pH 3.2) (A) and 1.5 mM ammonium formate in acetonitrile/methanol (95.5:0.5, v/v) (pH 3.2) (B) at flow rate of 250 µL/min. The mobile phase gradient ran from 10% A to 70% A over 7 min, ran from 70% A to 94.5% A over 2 min and was held at 94.5% A for 5 min, returning to 10% A over 2 min and held at 10% A for re-equilibration. The sample injection volume was 10 µL, the column temperature was set at 30 °C and the sample injector was maintained at 10 °C. Total run time per sample, including column cleaning and re-equilibration, was 25 min. The mass spectrometric analysis was performed using a TSQ Quantum Access (Thermo Fisher Scientific, Waltham, MA, USA) triple quadrupole mass spectrometer equipped with an electrospray ionization (ESI) interface operated in positive mode. The analytes were detected by tandem mass spectrometry (MS/MS) using multiple reaction monitoring (MRM). The LOQ value is ≤0.25 µM for all compounds, making this method suitable for the analysis of samples containing relatively low concentrations of the analytes, with a satisfactory precision as documented by the intra- and inter-day CVs of less than 10%. The method is linear in a wide range of concentrations (between 0 and 20 µM), with correlation coefficients greater than 0.99 and limit of detection (LOD) around 3–10 nm for all compounds.

### 2.6. Dot-Blot, Western Blot and Immunohistochemistry

Protein expression was evaluated by dot-blot, Western blot and immunohistochemistry techniques using specific antibodies against GSH (Santa Cruz Biotechnologies, Dallas, TX, USA), β-Actin, γH2AX and GAPDH (Cell Signalling, Leiden, The Netherlands) following standard protocols.

### 2.7. In Vitro Model of Protein S-Glutathionylation

An in vitro model using human umbilical vein endothelial cells (HUVEC) was implemented. The block glutathione reductase was achieved with a specific compound named 2-AAPA (Sigma-Aldrich, Darmstadt, Germany) at two different doses, 50 and 100 μM. Briefly, HUVEC were treated with 2-AAPA for 4 h in full M200 media (Thermo Fisher Scientific, Waltham, MA, USA). HUVEC were let recover for 24 h in fresh full media. At the end of the recovery time, GSH/GSSG levels, proteins and mRNA transcripts were evaluated.

### 2.8. Immunoprecipitation

Cells were lysed in 0.1% IGEPAL, 0.01% sodium dodecyl sulphate, 15 mM NaCl, 0.5% sodium deoxycholate and 0.1 mM EDTA (Sigma-Aldrich, Darmstadt, Germany). Five hundred micrograms of endothelial cell protein extract were pre-cleaned at 4 °C for 30 min with 0.5 μg of non-immune IgG coupled to 10 μL of protein G agarose beads (Bio-Rad, Hecules, CA, USA). Pre-cleaned extracts were centrifuged for 60 seconds at 300 × *g* and the supernatant was immunoprecipitated overnight at 4 °C with 10 μL of protein G agarose beads and 3 μg of GSH antibody (Santa Cruz Biotechnologies, Dallas, TX, USA) *per* 100 μg of protein. After washing three times with lysis buffer, the beads were boiled in reducing Laemmli buffer for 5 min and loaded onto SDS/polyacrylamide gel.

### 2.9. Reverse Transcription and Real-Time PCR

Extraction of RNA was performed from HUVEC using the Total RNA Purification Plus Kit (Norgen Biotek Corp., Thorold, ON, Canada). RNA was treated with DNAse to ensure purity and then quantified by Nanodrop. Two-step PCR amplification with TaqMan Reverse Transcription Reagent kit (Thermo Fisher Scientific, Waltham, MA, USA) was performed. Total RNA (1 μg) was converted into cDNA. The reference gene evaluation was performed with a predesigned 96-well plate panel for SYBR Green (Reference Gene H96, Bio-Rad, Hecules, CA, USA). Quantitative Real-Time PCR (qPCR) was performed on ABI Prism 7900 HT (Thermo Fisher Scientific, Waltham, MA, USA), according to the manufacturer’s instructions and analyses were performed using software SDS2.4 (Thermo Fisher Scientific, Waltham, MA, USA). Primers: *PECAM1* Fw (5’- CAG GCC CCA TTG TTC CC -3’); *PECAM1* Rv (5’- ATT GCT CTG GTC ACT TCT CC -3’); *CDH5* Fw (5’- GAT CAA GTC AAG CGT GAG TCG -3’); *CDH5* Rv (5’- AGC CTC TCA ATG GCG AAC AC -3’); *ACTA2* Fw (5’- AGA GTT ACG AGT TGC CTG ATG -3’); *ACTA2* Rv (5’- CTG TTG TAG GTG GTT TCA TGG A -3’); *GAPDH* Fw (5’- ACA TCG CTC AGA CAC CAT G -3’); *GAPDH* Rv (5’- TGT AGT TGA GGT CAA TGA AGG G -3’).

### 2.10. Statistical Analysis

The data were analyzed using IBM SPSS statistic software (version 22) and Graph Pad Prism software (version 7). Continuous variables were expressed as mean ± standard error (SEM). Between-group differences were evaluated by Student t-test, by one-way ANOVA with Bonferroni correction and by Pearson Chi-square test. A value of *p* < 0.05 was considered statistically significant.

## 3. Results

### 3.1. Patient Characteristics

The isolated CABG population included 58 patients. Their demographic, laboratory and clinical features are listed in Table 1. Fifty percent of patients (*n* = 29) had normal aortic valve morphology (No-AVSc group), while the remaining 50% (*n* = 29) was classified as aortic valve sclerosis morphology (AVSc group). The two groups were comparable for all studied variables, including age, hypertension, dyslipidaemia, diabetes mellitus, smoking habits, body mass index, New York Heart Association (NYHA) class, the severity of coronary artery disease, echocardiographic parameters and pharmacological treatments. In addition, the two groups also had comparable pre-operative C-reactive protein (CRP) levels.

### 3.2. Oxidative Stress and Endothelial Dysfunction

**Figure 1:**
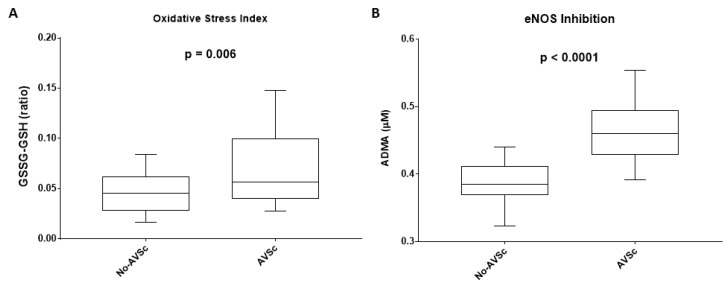
Imbalance of systemic glutathione homeostasis and endothelial dysfunction. (**A**) Box plot representing the ratio between the reduced (GSH) and the oxidized (GSSG) forms of glutathione in patients with normal aortic valve leaflet (No-AVSc; *n* = 29) and aortic valve sclerosis (AVSc; *n* = 29). (**B**) Box plot representing ADMA levels in patients with No-AVSc (*n* = 29) and AVSc (*n* = 29).

To evaluate systemic glutathione homeostasis, we measured the circulating levels of GSH and GSSG. Pre-operatively, GSSG/GSH ratio was significantly higher in AVSc patient’s group (0.070 ± 0.007) than No-AVSc patients (0.047 ± 0.004; *p* = 0.006, Figure 1A).

To assess endothelial dysfunction, we measured ADMA levels. Pre-operatively, ADMA concentration was significantly higher in AVSc patients (0.47 ± 0.009 μM) compared to No-AVSc patients (0.39 ± 0.007 μM; *p* < 0.0001, Figure 1B).

### 3.3. Systemic Inflammation Status

Since CRP pre-operative levels were similar between the two groups (No-AVSc = 2.01 ± 0.25 and AVSc = 2.31 ± 0.33), we considered CRP levels at other subsequent time points (at 3rd-day post-intervention, at 5th-day post-intervention and at discharge). We found that at 3rd-day after the intervention, CRP levels were significantly higher (+30.7, 95%CI: +3.9, +57.5; *p* = 0.02) in AVSc patients compared to No-AVSc ones (Supplemental Figure S1), indicating a possible different burst of inflammation caused by the intervention in these two groups. However, at 5th-day after the intervention and at discharge the levels between the two groups were comparable (Supplemental Figure S1).

### 3.4. Aortic Valve Protein S-Glutathionylation

**Figure 2:**
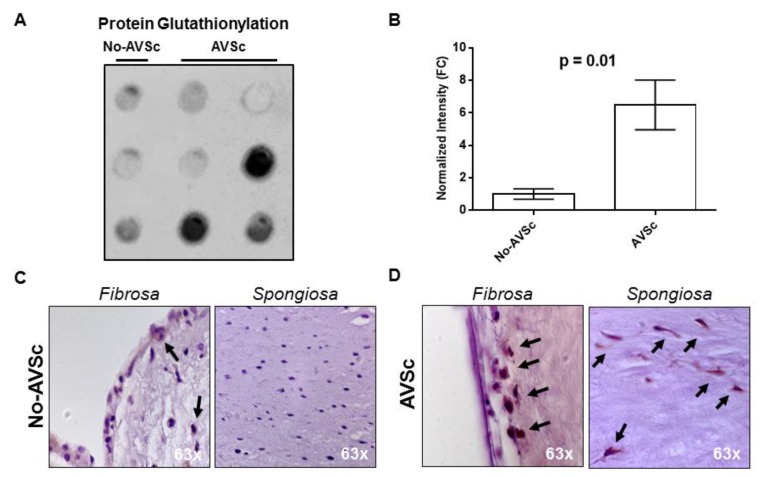
Aortic valve protein S-glutathionylation. (**A**,**B**) Dot-blot evaluation of total glutathione (GSH) expression in aortic valve leaflets (controls *n* = 3; AVSc *n* = 6) and relative quantification showed in the bar graph (dot-blot analyses using ImageJ; plugin: Dot-blot Analyzer v1.0). (**C**,**D**) Representative images showing histological analysis of human aortic valve in *Fibrosa* and *Spongiosa* layers. No-AVSc: patients with a normal aortic valve leaflet. AVSc: patients with aortic valve sclerosis. Black arrows indicate positive cells for GSH staining.

The systemic imbalance of glutathione homeostasis could also reflect a confined alteration leading to aberrant protein glutathionylation (Pr-SSG) in loco (i.e., in aortic valve leaflets). We evaluated this reversible post-translational modification in aortic valve whole protein extracts from patients that underwent concomitant CABG and aortic valve replacement (AVR) due to aortic valve insufficiency. Three specimens had normal morphology (No-AVSc), while six had AVSc. Dot-blot analysis revealed that AVSc specimens had significantly increased Pr-SSG levels (+6.5 ± 1.5) than No-AVSc (*p* = 0.01, Figure 2A,B). In addition, immunohistochemistry revealed that protein glutathionylation occurred in valve endothelial cells of both groups (No-AVSc and AVSc), while in valve interstitial cells Pr-SSG was present only in AVSc group (Figure 2C,D and Supplemental Figure S2).

### 3.5. In Vitro Model of Protein S-Glutathionylation

**Figure 3:**
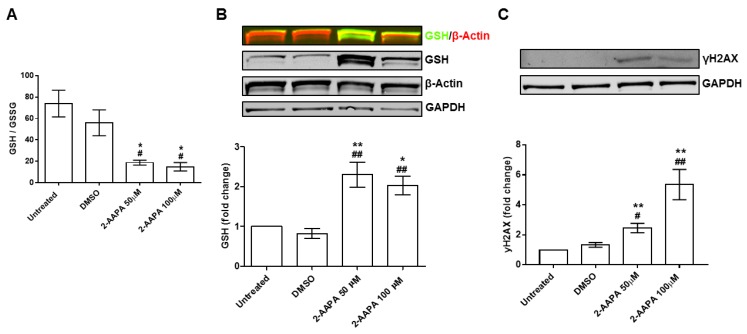
In vitro model of protein S-glutathionylation. (**A**) Bar graph representing the ratio between the reduced (GSH) and the oxidized (GSSG) form of glutathione (GSH/GSSG ratio) in human umbilical vein endothelial cells (HUVEC) after 4 h of treatment (2-AAPA 50 and 100 μM) and 24 h of recovery (*n* = 3). (**B**) Western blot representative images of HUVEC treated with 2-AAPA (50 and 100 μM) for 4 h and 24 h of recovery (*n* = 4); GSH is shown in green and β-actin is shown in red; Bar graph representing Western blot quantification by ImageJ. (* *p* < 0.05 vs. Untreated; ** *p* < 0.001 vs. Untreated; ## *p* < 0.001 vs. Dimethyl Sulfoxide (DMSO)). (**C**) Western blot evaluation of histone 2AX phosphorylation (γH2AX) in HUVEC after 2-AAPA (50 and 100 μM) treatment for 4 h and 24 h of recovery (*n* = 4); Bar graph representing Western blot quantification by ImageJ. (** *p* < 0.001 vs. Untreated; # *p* < 0.05 vs. DMSO; ## *p* < 0.01 vs. DMSO). Glyceraldehyde-3-Phosphate Dehydrogenase (GAPDH) has been used for normalization.

To study the effects of aberrant Pr-SSG as a trigger of endothelial damage, we implemented an in vitro model using HUVEC. As shown in Figure 3A, both 2-AAPA concentrations were able to drastically reduce the GSH/GSSG ratio (*p* < 0.05). As expected, the drop in GSH/GSSG ratio led to a significant increment of Pr-SSG. In particular, we noticed an increment of Pr-SSG by +2.3 ± 0.6 fold change (2-AAPA 50 μM) and by +2.1 ± 0.5 fold change (2-AAPA 100 μM) when compared to untreated cells or DMSO controls (*p* < 0.001, Figure 3B). Interestingly, the glutathionylated bands were at the same molecular weight of β-actin. Indeed, immunoprecipitation confirmed that β-actin was actually the protein that underwent glutathionylation (Supplemental Figure S3). In addition, we noticed that 2-AAPA, at both concentrations, induced the phosphorylation of histone 2AX (γH2AX), indicating DNA double-strand brakes (Figure 3C).

### 3.6. Protein S-Glutathionylation and Endothelial-to-Mesenchymal Transition

**Figure 4:**
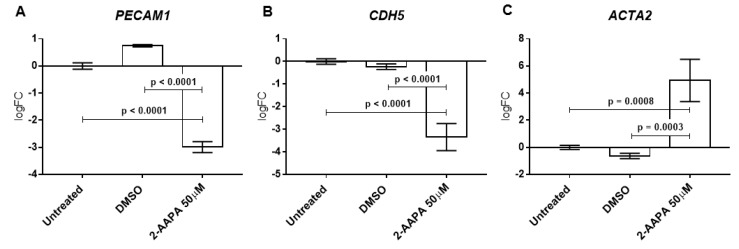
Protein S-glutathionylation and endothelial-to-mesenchymal transition. Quantitative Polymerase Chain Reaction (qPCR) of the gene encoding endothelial markers (**A**) CD31, (**B**) VE-cadherin and mesenchymal marker (**C**) *ACTA2* after 4 h of 2-AAPA (50 μM) treatment and 48 h of recovery (*n* = 3). qPCR data are expressed as log2 fold change (logFC) in comparison to untreated cells.

To evaluate the involvement of Pr-SSG in the endothelial-to-mesenchymal transition (EndMT), we treated the HUVECs with the minimum dose of 2-AAPA that caused Pr-SSG and DNA damage (50 μM). We, therefore, assessed endothelial specific genes, such as platelet and endothelial cell adhesion molecule 1 (*PECAM1*) and cadherin 5 (*CDH5*) and activated fibroblast specific gene, such as alpha-smooth muscle actin 2 (*ACTA2*). First, we checked for the best housekeeping gene in our experimental conditions with the implementation of four different algorithms. The comprehensive gene stability analysis allowed us to recognized glyceraldehyde-3-phosphate dehydrogenase (*GAPDH*) as the most stable gene (Supplemental Figure S4). Finally, as shown in Figure 4, we found significant downregulation of both *PECAM1* and *CDH5* and a significant upregulation of *ACTA2* in HUVEC treated with 2-AAPA in comparison to untreated and DMSO treated cells (all *p* < 0.001).

## 4. Discussion

In this study, we have shown for the first time that patients with overt atherosclerosis and AVSc had systemic glutathione homeostasis imbalance. The glutathione imbalance was associated with an elevated Pr-SSG in fibrotic aortic valves (i.e., AVSc) and in vitro experiments showed that increment in Pr-SSG induced DNA damage with direct consequences on EndMT.

AVSc is present in approximately 30% of people with more than 65 years of age [19,20] and the AVSc prevalence could reach up to 75% in patients with coronary atherosclerosis [21,22]. Previous reports have noted that AVSc was an independent predictor of coronary artery disease (CAD) [23] and it was associated with a high rate of coronary occlusion [21]. In addition, AVSc is an important predictor of myocardial infarction in patients with known CAD [24], as well as major cardiovascular and all-cause mortality [6,19,25]. Hence, in the present study, we used patients with overt atherosclerosis due to a high chance to find subjects with AVSc [26,27,28,29].

Several clinical studies evidenced an association between AVSc and coronary atherosclerosis with systemic endothelial dysfunction measured by flow-mediated dilation, carotid intima-media thickness and pulse wave velocity [7,30,31]. Indeed, it has been hypothesized that a possible trigger of AVSc is represented by endothelial dysfunction [32]. The mechanisms by which the aortic valve endothelium influences the development of AVSc is still under investigation.

In this study, we showed that AVSc is characterized by an imbalance of the glutathione system that leads to an aberrant protein glutathionylation and an increment in ADMA levels. Similar results were obtained in patients with AS who required AVR [33,34,35].

In calcific aortic valves, it has been shown that the dysfunction of the antioxidant mechanisms contributes to the increment of oxidative stress levels and to the dysregulation of post-translational modifications (such as Pr-SSG) with direct consequences on the nitric oxide synthase activity (eNOS) [36]. Indeed, eNOS could undergo the so-called eNOS uncoupling due to increased oxidative stress levels and to consequent Pr-SSG. The eNOS uncoupling represents the switch from its classical NO synthase function to that of an NADPH-dependent oxidase generating O_2_^•−^ [37]. However, we were not able to evaluate the eNOS uncoupling since the only evident glutathionylated protein was β-actin.

Another feature of AS is the EndMT that was observed in concomitance to osteogenic marker expression [38]. Indeed, valve endothelial cells are able to regulate valve interstitial cell phenotype and when EndMT occurs this homeostasis is lost [39]. The EndMT is the complex biological process that drives the remodelling of the underlying tissues [40]. It is characterized by loss of endothelial markers and acquisition of mesenchymal ones [41]. In our study, glutathione reductase inhibition led to decreased VE-cadherin gene expression levels (endothelial marker) and to an increment of *ACTA2* expression (mesenchymal marker), suggesting that aberrant Pr-SSG could be implicated in EndMT. In this context, it has been shown that endothelial cells, in response to stimuli that promote arteriosclerosis, contribute to vascular calcification via EndMT [42]. Hereof, our results support that dysregulation in the glutathione system leads to an excess in Pr-SSG and DNA damage, with direct consequences on EndMT induction. It has to be pointed out that the used 2-AAPA concentrations are based on several in vitro studies, showing that both 50 and 100 μM are able to completely inhibit glutathione reductase [43,44,45].

Finally, in our study, we found that AVSc prevalence in patients with atherosclerosis was associated with endothelial damage and oxidative stress but not with the inflammatory status of the patients. Inflammation was recognized as an important pathological mechanism of CAVS development [46]. A number of studies show the significant association of increased CRP levels, known marker of inflammation, with AS [47,48,49]. Moreover, high CRP expression in aortic valve leaflet correlated with serum high CRP level in AS patients [50]. Plasma hs-CRP levels were also related to faster AS progression [51]. However, Jeevanantham et al. [52] suggested that hs-CRP correlated with an earlier stage of CAVS but not with AS severity. Nevertheless, according to a large population-based study (5621 participants), where approximately 9% of subjects with AVSc progressed to AS over a 5-year follow-up, CRP was not associated with either the presence nor the progression of CAVS [53]. In addition, the association of AVSc with tissue NO resistance and not with systemic inflammation, measured by hs-CRP, was also recently reported [54]. In line with this, in our study, we did not find any association with circulating CRP levels and AVSc presence.

## 5. Conclusions

In conclusion, our results suggest that oxidative stress leading to protein glutathionylation could represent a key point on the onset and progression of the aortic valve sclerosis. Thus, the reduction of protein glutathionylation, by the restoration of glutathione balance or by the activation of enzymes able to remove this post-translational modification such as glutaredoxins or thioredoxins, may shed light on new therapeutic targets to slow down or even halt calcific aortic valve stenosis.

## Figures and Tables

**Table 1:** Patient Characteristics.

Variable	CABG (*n* = 29)	CABG + AVSc (*n* = 29)	*p* Value
Age, years	62.2 ± 6.2	65.2 ± 8.4	0.133
Male sex, *n* (%)	29 (100)	29 (100)	1.000
Diabetes, *n* (%)	7 (24)	5 (17)	0.525
Hypertension, *n* (%)	17 (59)	22 (76)	0.168
Dyslipidemia, *n* (%)	22 (76)	19 (65.5)	0.396
Current Smoking, *n* (%)	3 (10)	7 (24)	0.171
Ex-Smokers, *n* (%)	15 (52)	13 (45)	0.607
Body mass index, kg/m^2^	26.7 ± 2.9	27.8 ± 3.6	0.156
Creatinine, mg/dL	0.91 ± 0.12	0.94 ± 0.17	0.411
C-reactive protein, mg/L	2.61 ± 2.56	2.73 ± 2.14	0.853
*New York Heart Association (NYHA) class*			
I	10 (34)	11 (38)	1.000
II	16 (56)	12 (41)	0.593
III	3 (10)	6 (21)	0.470
IV	-	-	-
3-Vessels coronary disease, *n* (%)	20 (69)	19 (65.5)	0.784
Logistic EuroSCORE	1.93 ± 1.79	2.68 ± 2.14	0.160
*Echocardiography*			
Left ventricle ejection fraction, *n* (%)	61.3 ± 10.1	57.9 ± 10.1	0.210
LV hypertrophy index, mm	0.35 ± 0.13	0.41 ± 0.12	0.134
Max. aortic velocity, m/s	0.99 ± 0.54	1.23 ± 0.59	0.100
Max. aortic gradient, mmHg	5.14 ± 3.16	7.55 ± 6.79	0.090
*Therapies*			
Antiplatelets, *n* (%)	21 (72)	18 (62)	0.410
Angiotensin receptor blockers, *n* (%)	5 (17)	6 (21)	0.743
Converting enzyme inhibitors, *n* (%)	8 (28)	11 (38)	0.410
Calcium channel blockers, *n* (%)	9 (31)	9 (31)	1.000
Beta-blockers, *n* (%)	19 (65.5)	19 (65.5)	1.000
Nitrates, *n* (%)	6 (21)	10 (34.5)	0.248
Statins, *n* (%)	18 (62)	19 (65.5)	0.789

The values are presented as the number of patients (*n*) with the percentage in brackets or mean ± standard error.

## Supplementary Materials

The supplementary materials are available online at https://www.mdpi.com/2077-0383/8/4/552/s1.
