# Supplementary material for: Impact of Oxidative Stress and Protein S-Glutathionylation in Aortic Valve Sclerosis Patients with Overt Atherosclerosis
Source: J Clin Med. 2019 Apr 24;8(4):552. doi: 10.3390/jcm8040552 (PMC6517913; doi:10.3390/jcm8040552)
Supplement: Supplementary file 1 [file jcm-08-00552-s001.pdf]

Supplemental Figure S1.

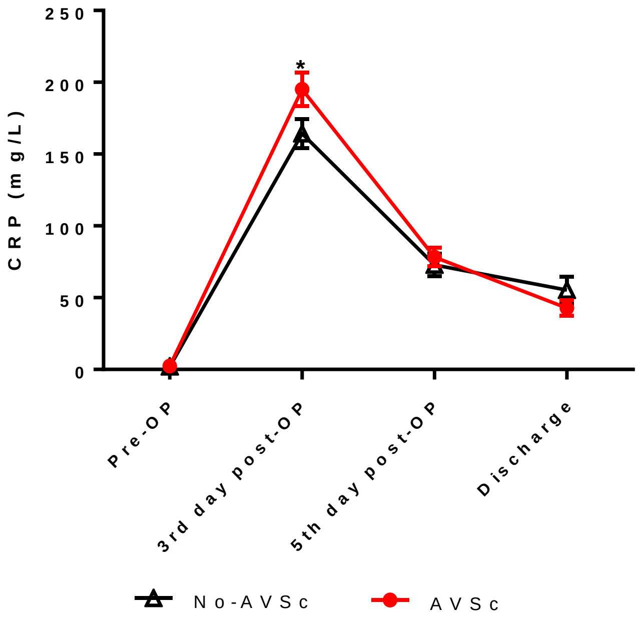

Systemic inflammation status. Graph representing C-reactive protein (CRP) levels measured in clinical routine preoperatively, at 3rd-day post-surgery, at 5th-day post-surgery, and at discharge. Black line denotes patients with normal aortic valve leaflets (No-AVSc; n = 29), while red line denotes patients with increase aortic valve leaflet thickness (AVSc; n = 29). * $p < 0.05$.

Supplemental Figure S2.

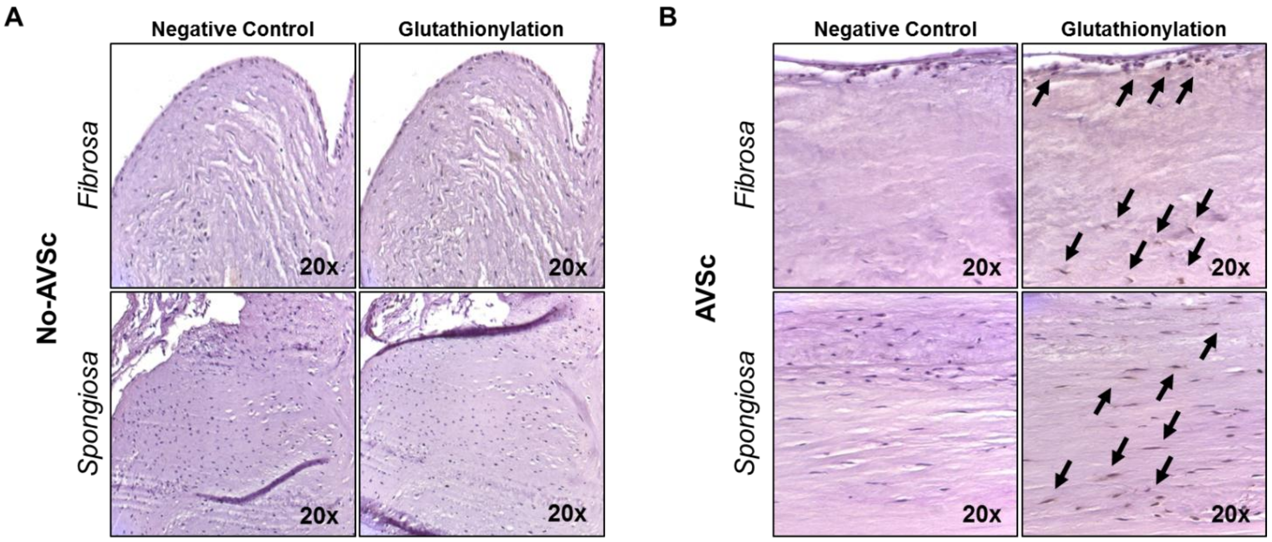

Protein S-glutathionylation in aortic valve leaflets. (**A-B**) Immunohistochemistry showing glutathione (GSH) expression in *Fibrosa* and *Spongiosa* layers of the aortic valve (**A**) in patients with normal aortic valve leaflets (No-AVSc) and (**B**) in patients with increased aortic valve leaflets thickness (AVSc). Black arrows indicate positive cells for GSH staining.

Supplemental Figure S3.

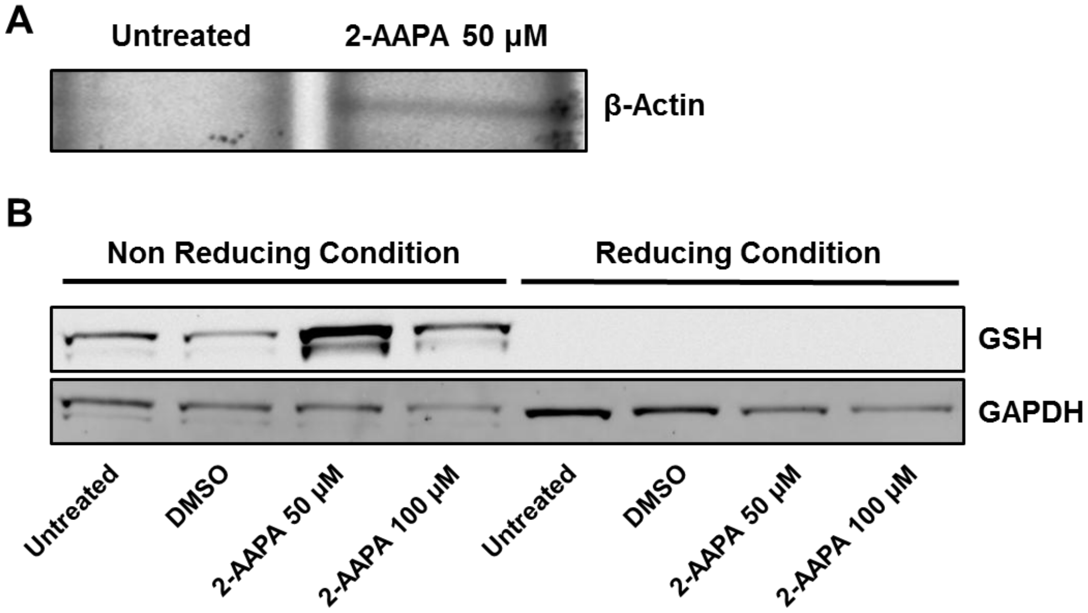

S-Glutathionylation of β-actin. (**A**) Immunoprecipitation of glutathione (GSH) and Western blot for β-actin in HUVEC treated with 2-AAPA 50 µM for 4 hours and 24 hours of recovery. (**B**) Western blot showing GSH in non-reducing and in reducing conditions in HUVEC untreated, treated with DMSO, 2-AAPA 50 µM, and 2-AAPA 100 µM.

Supplemental Figure S4.

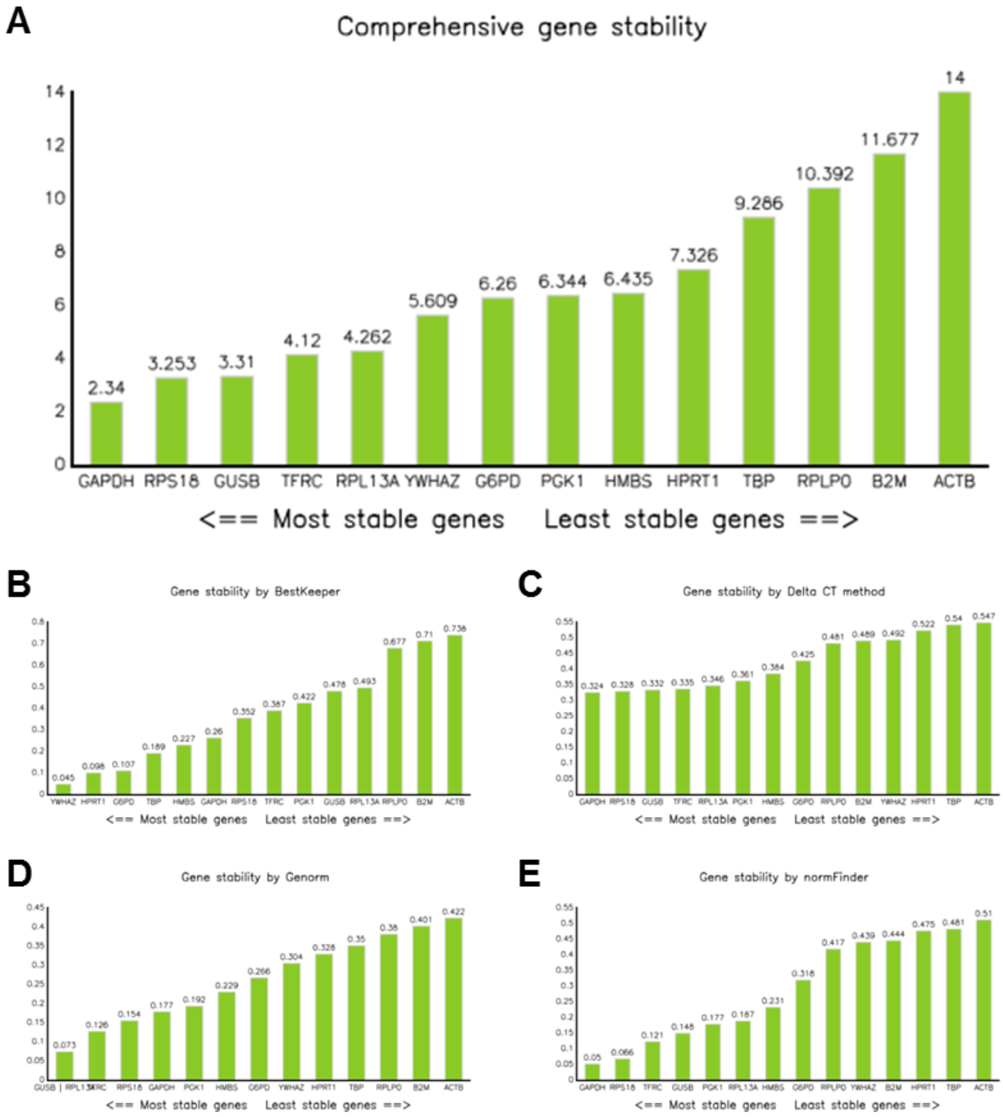

Comprehensive gene stability. (**A**) Histograms showing the evaluation of comprehensive gene stability comparing untreated *vs.* 2-AAPA 50 μM cells (n = 3) assessed by following different algorithm: (**B**) Bestkeeper; (**C**) Delta CT method; (**D**) Genorm; and (**E**) Normfinder.
